# The Effect of Thermal Exfoliation Temperature on the Structure and Supercapacitive Performance of Graphene Nanosheets

**DOI:** 10.1007/s40820-014-0014-4

**Published:** 2014-10-31

**Authors:** Haiyang Xian, Tongjiang Peng, Hongjuan Sun, Jiande Wang

**Affiliations:** 1 Institute of Mineral Materials & Application, Southwest University of Science and Technology, Mianyang, 621010 People’s Republic of China; 2 School of Material Science and Engineering, Southwest University of Science and Technology, Mianyang, 621010 People’s Republic of China

**Keywords:** Graphene nanosheets, Thermal exfoliation temperature, Supercapacitive performance

## Abstract

Graphene nanosheets (GSs) were prepared from graphite oxide by thermal exfoliation method. The effect of thermal exfoliation temperature on the structure and supercapacitive performance of GSs has been investigated. The results show that the GSs with pore sizes center around 4.0 nm. With an increase of thermal reduction temperature, the number of stacking layers and the structure disorder degree increase, while the oxygen-containing groups content, BET surface area, and electrical resistivity of GSs decrease. The results indicate that 673 K is the preferable thermal exfoliation temperature to acquire good supercapacitive performance. In this case, the GSs have the best supercapacitive performance (233.1 F g^−1^) in a 6 mol L^−1^ KOH electrolyte. The prepared GSs at the preferable thermal exfoliation temperature have good rate performance and cycle stability.

## Introduction

Supercapacitor, which includes electrical double layer capacitor (EDLC) and pseudocapacitor, is a new type of energy storage device [1]. It is widely used for energy capture and storage applications because it has greater power density and longer cycling life than ordinary batteries and higher energy density than conventional capacitor [2]. The supercapacitive performance depends on the electrode materials. So far, carbon [3], conducting polymers [4, 5], transition metal oxides (such as RuO_2_ [6], MnO_2_ [7], and NiO [8]), and their composites [9–11] are the main materials used for the electrodes of supercapacitors. Carbon-based materials (such as activated carbon [2], carbon fibers [12], carbon nanotubes [13], carbon aerogels [14], and graphene [15]) have become the hot ones because of their high specific capacitance, long cycle life, and structural diversity.

Graphene, a two-dimensional graphite plane with one-atomic thickness, has attracted tremendous attention due to its unique physical, chemical, and mechanical properties [16]. At present, there are kinds of methods to fabricate graphene, such as mechanical exfoliation [16], reduction of graphite oxide, GO [17], liquid-phase exfoliation [18], and epitaxial growth involving the chemical vapor deposition [19] as well as the thermal decomposition of SiC [20]. Among these methods, the reduction of GO is a potential way for industrial application, because of its cheap raw materials, low demand for equipment, simple process, and mass production. There are three main kinds of reduction methods: chemical reduction [21, 22], electrochemical reduction [23], and thermal exfoliation reduction [24]. Although the chemical reduction method with kinds of reducing agents is used in a wide range of applications, it is similar to the electrochemical method for some disadvantages, such as they are time consuming, and also the reducing agents are toxic. Thus, the thermal exfoliation reduction is a potential method for the industrial scale production. Graphene from thermal exfoliation reduction method still contains kinds of oxygen-containing groups, such as hydroxyl (C–OH), epoxy (C–O–C), and carboxyl groups (COOH). Graphene with oxygen-containing groups can produce faradaic pseudocapacitance, which is helpful to improve the electrochemical performance of graphene [25]. Recently, graphene, prepared from thermal exfoliation for supercapacitors, has been intensively investigated. And also the thermal reduction mechanism was investigated [26]. Compared with the heating of nanodiamond and the decomposition of camphor over nickel nanoparticles methods, the graphene, prepared by thermal exfoliation (over 1,273 K) of GO, has the highest specific capacitance of about 117 F g^−1^ in a 1 M H_2_SO_4_ aqueous electrolyte [27], and also the graphene has high specific surface area and the specific capacitance of 150 F g^−1^ under specific current of 0.1 A g^−1^ for 500 cycles of charge/discharge in a KOH aqueous solution (30 wt%) electrolyte [28].

Although many works are based on the method of thermal exfoliation of GO, the effect of thermal reduction temperature on the structure and capacitive performance of graphene has not been reported before. So it will be helpful if we find out what happens during the thermal exfoliation process. In the present work, graphene nanosheets (GSs) were prepared by thermal exfoliation method. The effect of thermal exfoliation temperature on the structure and supercapacitive performance has also been investigated.

## Experimental

### Sample Preparation

GO was prepared by the simplified Hummers method that we always use in our group. Firstly, natural flake graphite powder (2 g, ~200 mesh, from the concentrating mill of Huangtuyao graphite deposit in the Nei Mongol Autonomous Region, China, designated as XHG) was added to concentrated H_2_SO_4_ (95–98 %, 46 mL), and the mixture was cooled down to 0 °C using an ice bath, and then the KMnO_4_ powder (10 g) was added slowly to keep the reaction temperature below 15 °C for 0.5 h. Secondly, the reaction mixture was heated to 35 °C and stirred for 2 h, at which ultrapure water was added slowly, giving rise to a pronounced exothermal effect up to 70 °C. The reaction mixture was stirred for 30 min, then 5 % of H_2_O_2_ was added until no gas produced, and, finally, the mixture was deposited for 12 h, until the supernatant being decanted away. The remaining solid material was then washed with ultrapure water and deposited again, this process being repeated until the pH was neutral. The reaction product (GO) was dried at 60 °C for 24 h.

GSs were obtained after the treated GO was put into tube furnace heated to given temperature (nitrogen protection, 373, 473, 673, 873, and 1073 K) for 30 min. The sample collected after the heating is designated as XHGS-*n*, where “n” denoted the corresponding heating temperature such as XHGS-673.

### Characterization of GSs

X-ray diffraction (XRD) measurements were conducted on a PANalytical X’Pert PRO multifunctional powder diffractometer. The radiation frequency used was the K αl line from Cu (0.15406 nm), with a power supply 40 kV and 40 mA. Fourier transform infrared (FT-IR) spectra of GO and XHGS-*n* samples were recorded at room temperature using a transmission mode with a KBr tableting and a Nicolet 5700 FT-IR spectrometer. Raman spectra were recorded on Renishaw InVia Raman Microprobe using a 514.5 nm Ar^+^ laser. The morphologies of the samples were observed by scanning electron microscope (SEM, ZEISS Ultra 55) and high-resolution transmission electron microscope (HRTEM, Zeiss Libra 200FE). The samples for HRTEM were prepared by dispersing the products in ethanol with an ultrasonic bath for 30 min and then a few drops of the resulting suspension were placed on a copper grid. Nitrogen adsorption–desorption isotherms were measured at 77 K using a Quantachrome Autosorb-1MP analyzer.

### Preparation of Electrodes and Electrochemical Test

The working electrodes were prepared by mixing the GSs with acetylene black and polytetrafluoroethylene in a weight ratio of 85:10:5, the resulting mixture was pressed onto nickel foam (1 cm × 3 cm) under 10 MPa for 30 s. The typical mass and dimensions of the working electrodes are about 2–3 mg and 1 cm^2^.

The supercapacitive performance of the GSs were determined in a three-electrode test cell, which contained a platinum foil electrode (1.5 cm × 1.5 cm) as a counter electrode, a Hg/HgO as reference electrode, and 6 mol L^−1^ KOH aqueous solutions. The cyclic voltammetry (CV) and galvanostatic charge/discharge (GCD) test were carried out on a CHI660E electrochemical work station at room temperature. The electrochemical impedance spectroscopy (EIS) was carried out on a PGSTAT 302N Autolab electrochemical work station. For the CV measurements, the sweep rate ranged from 5 to 50 mV s^−1^ within a potential range of −0.8 to 0.2 V. For the GCD measurements, the potential ranged from −0.8 to 0.1 V and the current density from 0.5 to 5.0 A g^−1^. For the EIS measurements, the perturbation potential was 5 mV and the frequency range was from 10 mHz to 100 kHz.

## Results and Discussion

### Morphologies and Pore Characteristics of GSs

**Fig. 1:**
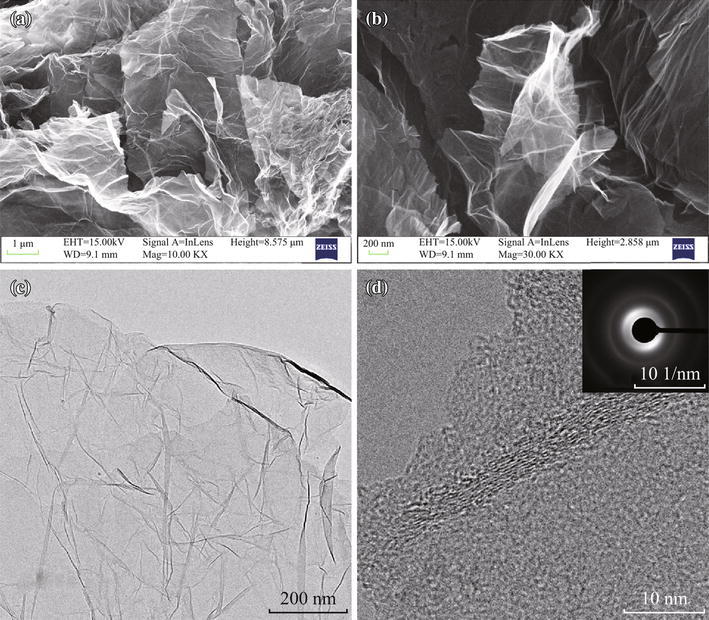
SEM images of XHGS-673 (**a**, **b**), HRTEM images of XHGS-673 (**c**, **d**), and the *inset* of **d** shows the corresponding SAED pattern

The morphologies of the obtained XHGS-673 were observed by SEM and HRTEM, and their images are shown in Fig. 1. Figure 1a exhibits the SEM image of XHGS-673 agglomerate, consisting of almost transparent GSs with thin wrinkled structure that graphene owns intrinsically [29]. Figure 1b is a SEM magnification image for an individual graphene sheet. Figure 1c shows the TEM image of XHGS-673, the thin wrinkled structure of GSs is displayed similar to the SEM image. Figure 1d shows the disordered graphene layers in XHGS-673, which is attributed to the disruption of the planar *sp*^2^ carbon by the introduction of *sp*^3^ carbon upon oxidation and heating process. The width of the wrinkle stripes indicates that XHGS-673 is composed of approximately five–seven wrinkled monolayer graphene. The selected area electron diffraction (SAED) pattern in the inset of Fig. 1d exhibits an annulus pattern, indicating the loss of long-range ordering between the graphene sheets. Similar patterns are also observed from XHGS-*n* at other temperature.

**Fig. 2:**
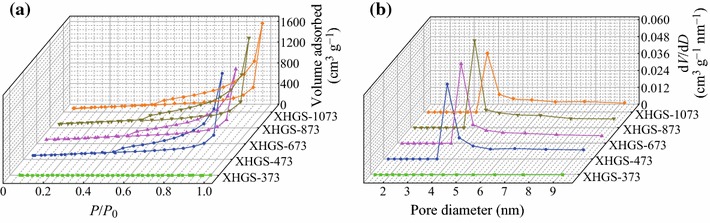
**a** N_2_ adsorption–desorption isotherms of graphene nanosheets and **b** BJH pore size distributions from adsorption branches for graphene nanosheets

Figure 2 displays the nitrogen adsorption–desorption isotherms and pore size distribution curves calculated by BJH methods, respectively. In Fig. 2a, except XHGS-373, other GSs all exhibit typical IV shapes, indicating their mesoporous characteristics. The BET surface area values for each sample are listed in Table 1. BJH analysis for pore diameter distribution is shown in Fig. 2b, the pore sizes of the samples all mainly center around 4.0 nm. It is well known that the uniform pore sizes in the range of 3–5 nm are required to improve the capacitance in EDLCs [30].

**Table 1:** Some properties of XHG and XHGS-*n*

Samples	*d*_002_ (nm)	*L*_c_ (nm)	Number of stacking layers	*I*_D_/*I*_G_	Resistivity (Ω m)	BET surface area (m^2^ g^−1^)
XHG	0.336	−	−	−	1.3 × 10^−6^	−
XHGS-373	0.775	4.2	5	1.45	8.3 × 10^−4^	−
XHGS-473	0.363	2.1	6	1.80	9.6 × 10^−5^	344.29
XHGS-673	0.361	2.4	7	2.00	6.1 × 10^−5^	374.40
XHGS-873	0.339	4.0	12	2.21	4.8 × 10^−5^	372.09
XHGS-1073	0.337	4.5	14	2.25	3.7 × 10^−5^	359.57

### Structures of GSs

**Fig. 3:**
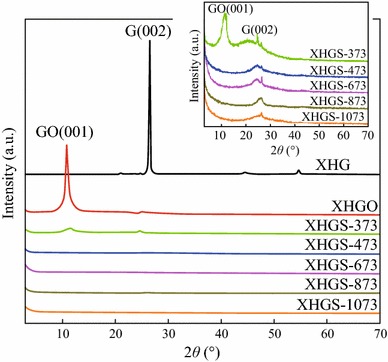
XRD patterns of XHG, XHGO, and XHGS-*n* samples, the *inset* is the enlarged map of the XHGS-*n* samples

The obvious structural changes of natural flake graphite (XHG) before and after the oxidation and heating process were investigated by XRD measurement, and the XRD patterns of XHG, XHGO, and XHGS-*n* samples are shown in Fig. 3. The G(002) diffraction peaks of XHGS-*n* become obviously weak and dispersive compared with that of XHG, indicating the graphite crystalline (XHG) has changed into disordered (a graphite-like) structure through the oxidation and high temperature (473, 673, 873, and 1073 K) heating treatments, while the low heating temperature (373 K) leads to the structure of XHGS-373 which is similar to that of XHGO. It can be seen that with the increase of heating temperatures (373, 473, 673, 873, and 1073 K), the interlayer spacing value of G(002) plane (*d*_002_) exhibits decreases gradually, which can be ascribed to the increase of graphitization degree of XHGS-*n*. The crystallite parameters *L*_c_ of the XHGS-*n* were calculated from the peak position, the half-height width of the G(002) peak using the Scherrer equation [31]:

1 $$L_{\text{c}}=\frac{K\lambda }{\beta \cos\theta_{002}},$$

where *K* is constant dependent on crystallite shape (0.89), *λ* is wavelength of Cu Kα1 radiation, *β* is the half-height width of the G(002), and *θ*_002_ is angle between the incident and scattered X-ray from G(002). The number of stacking layers is calculated by the equation:

2 $$n=L_{\text{c}}/\;d_{002}$$

The calculated results are presented in Table 1. It can be seen that with the increase of heating temperature, the *L*_c_ decreases firstly and then increases, and the number of stacking layers of the XHGS-*n* increases gradually.

**Fig. 4:**
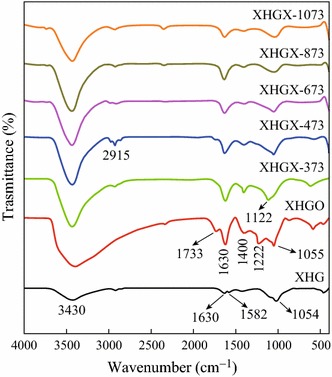
FT-IR spectra of XHG, XHGO, and XHGS-*n* samples

The FT-IR spectra of XHG, XHGO, and XHGS-*n* are shown in Fig. 4. The FT-IR spectrum of XHG shows a stretching vibration and a bending vibration of absorbed H_2_O at 3,430 and 1,630 cm^−1^, respectively, and also a skeletal vibration of C=C in hexagonal ring at 1,582 cm^−1^, corresponding to the planar *sp*^2^ structure that graphite owns intrinsically. After oxidation treatment, a series of peaks appear in the FT-IR spectrum of XHGO. The peaks at 1733, 1400, 1222, and 1122 cm^−1^ correspond to the stretching vibration of C=O, the bending vibration of C–OH, the stretching vibration of C–O in the carboxy group, and the stretching vibration of C–O–C, respectively. After the heating process, with the increase of heating temperature, all the FT-IR absorbance peaks of oxygen-containing groups weaken gradually, suggesting that the oxygen-containing groups were dislodged from the structure of GO after the thermal reduction process.

**Fig. 5:**
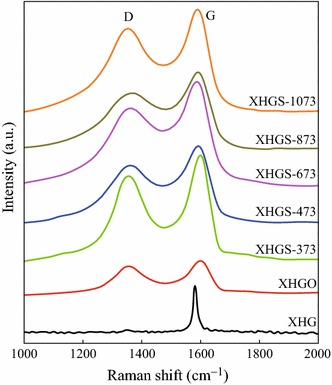
Raman spectra of XHG, XHGO, and XHGS-*n* samples

Raman technology was performed to characterize the disorder before and after the oxidation and heating process. The Raman spectra of XHG, XHGO, and XHGS-*n* samples are shown in Fig. 5. The G-band in the Raman spectra, arising from the zone center E_2_g mode, corresponds to the ordered *sp*^2^ bonded carbon, while the D-band is ascribed to the edges and other defects [32]. After oxidation and rapid heat process, the G-band of each XHGS-*n* sample gets broad and weakened, while D-band becomes increasingly strong. The area ratios of D-band to G-band (*I*_D_/*I*_G_) are listed in Table 1, indicating the degree of disorder for each XHGS-*n* sample, respectively. With the increase of heating temperature, the degree of disorder exhibits increase, indicating that higher temperature leads to more disorder in the structure of GSs. The electrical resistivity of the GSs is also listed in Table 1. The electrical resistivity of the GSs decrease with the heating temperature because of higher temperature leads to less oxygen-containing groups in the structure, and less oxygen-containing groups leads to better electroconductibility [33], which is complied with the result from FT-IR test.

### Supercapacitive Performance

**Fig. 6:**
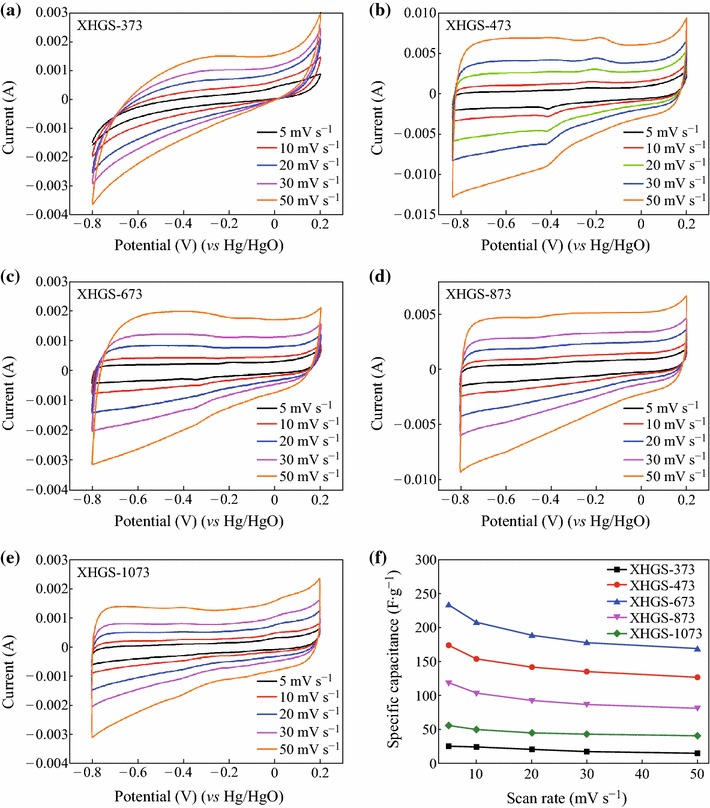
Cyclic voltammograms of XHGS-*n* (**a**–**e**) at different scan rates (from 5 to 50 mV s^−1^) and comparison of specific capacitance values with increasing scan rates for XHGS-*n* (**f**)

CV measurements were carried out to analyze the supercapacitive performance of the XHGS-*n*. Figure 6 presents the CV curves of XHGS-373 (a), XHGS-473 (b), XHGS-673 (c), XHGS-873 (d), and XHGS-1073 (e) at scan rates varying from 5 to 50 mV s^−1^. The CV curves of XHGS-373 shows clinographic curves, suggesting poor electrical conductivity for XHGS-373, while other samples show typical rectangular curves without obvious oxygen and hydrogen evolution peaks which are due to the oxygen-containing groups except for XHGS-473. With the increase of heating temperature, the rectangular curves of XHGS-*n* are closer to the ideal rectangular shape, indicating that the supercapacitive performance become well due to the removal of the oxygen-containing groups. The specific capacitance (*C*_CV_) is calculated by Eq. (3):

3 $$C_{\text{CV}}=\frac{\int i\text{d}V}{2m\cdot \upsilon \cdot \Delta V},$$

where ∫*i*d*V* is the integral area of the CV curve, *m* is the mass of active material in the electrode, *υ* is scan rate, and Δ*V* is the potential window. The specific capacitances at different scan rates are presented in Fig. 6f. The specific capacity values of the XHGS-373 and XHGS-1073 are obviously lower than those of XHGS-473, XHGS-673, and XHGS-873 at different scan rates from 5 to 50 mV s^−1^. Under the scan rates from 5 to 50 mV s^−1^, the retention specific capacity values of XHGS-373, XHGS-473, XHGS-673, XHGS-873, and XHGS-1073 are 59.6, 72.8, 72.5, 69.2, and 74.2 %, respectively. The specific capacity value of XHGS-673 is the highest among all the samples, which can reach 233.1 F g^−1^ at the scan rate of 5 mV s^−1^ due to the heating temperature.

According to the structure analysis of the XHGS-*n*, with the increase of the heating temperature, the *sp*^3^-bonded carbon recovers to *sp*^2^-bonded carbon which leads to the increase of the electrical conductivity, so that the specific capacity values increase with the heating temperature. Follow to this law, the specific capacity values of XHGS-873 and XHGS-1073 should be higher than that of XHGS-673, but the result is the opposite. The specific capacity values of XHGS-873 and XHGS-1073 are 118.8 and 55.6 F g^−1^, respectively, at the scan rate of 5 mV s^−1^, indicating that the specific capacity value decrease with the heating temperature from 673 to 1,073 K. According to the analysis results of the XRD patterns for XHGS-*n*, the number of stacking layers increase with the heating temperature, that is why the specific capacity values decrease with the heating temperature.

**Fig. 7:**
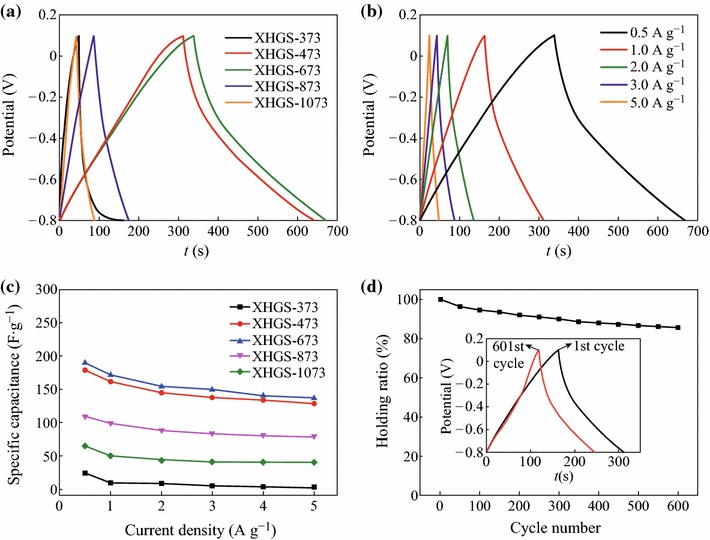
**a** The first galvanostatic charge/discharge cycling curves for XHGS-*n* at a current density of 0.5 A g^−1^, **b** galvanostatic charge/discharge cycling curves for XHGS-673 at various current densities from 0.5 to 5.0 A g^−1^, **c** the specific capacitances of XHGS-*n* electrodes at various current densities from 0.5 to 5.0 A g^−1^, and **d** capacitance curves of XHGS-673 under a current density of 0.5 A g^−1^, the *inset* of **d** shows the comparison of galvanostatic charge/discharge curves between 1st and 601st cycles for XHGS-673

The first GCD cycling curves for XHGS-*n* at a current density of 0.5 A g^−1^ are shown in Fig. 7a. All the curves deviate from regular triangular shape to certain extent. The oxygen-containing groups remaining in the structure of XHGS-*n* from the oxidation process cause redox reaction during charge/discharge process, resulting in the deviation of the cycling curves. Figure 7b shows the GCD cycling curves for XHGS-673 at the current density from 0.5 to 5.0 A g^−1^. With the increase of the current density, the triangular shape is closer to the ideal isosceles triangle, suggesting that the supercapacitive performance become well at high current density. Similar regulars are also observed from XHGS-*n* at other temperature.

The specific capacitance (*C*_GCD_) is calculated from the GCD curves by Eq. (2):

4 $$C_{\text{GCD}}=\frac{I\cdot \Delta t}{m\cdot \Delta V},$$

where *I* is the current of charge–discharge, Δ*t* is the time of discharge, *m* is the mass of active materials in the working electrode, and Δ*V* is 0.9 V. Figure 7c shows the specific capacitances of XHGS-*n* electrodes at various current densities from 0.5 to 5.0 A g^−1^. At 0.5 A g^−1^, XHGS-373, XHGS-473, XHGS-673, XHGS-873, and XHGS-1073 electrodes show stable *C*_GCD_ of 23.4, 178.9, 189.1, 109.1, and 64.7 F g^−1^, respectively. Although differences are existed between *C*_GCD_ and *C*_CV_, the *C*_GCD_ values change tendency with the heating temperature is the same as *C*_CV_. It can be speculated that the number of stacking layers and the content of oxygen-containing groups are the key factors which are caused by the differences of the heating temperature. The specific capacity value decrease with the increase of the stacking layer number and the oxygen-containing groups in the GSs exhibits as faradaic pseudocapacitor which can increase the specific capacity. CV and GCD measurements results suggest that 673 K is the best temperature point for their supercapacitive performance. Figure 7d displays the capacitance versus cycle number curves of XHGS-673 under the current density of 0.5 A g^−1^, the inset of Fig. 7d shows the comparison of GCD curves between 1st and 601st cycles for XHGS-673. It can be seen that stable capacitances with the holding rate of 85.8 % after 600 cycles. The good cycle performances of XHGS-673 imply its stable energy storage processes during long cycle charging/discharging.

**Fig. 8:**
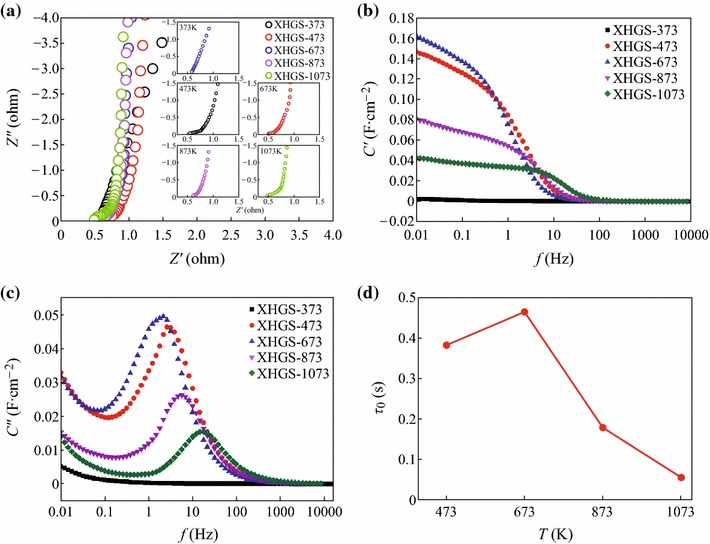
**a** Nyquist impedance plots, **b** the relationship between the real capacitance and frequency, **c** the relationship between the imaginary capacitance and frequency, and **d** the variation of the relaxation time with the heating temperature

EIS as a powerful technique was further employed to monitor the supercapacitive performance of XHGS-*n* electrodes. Figure 8a shows the Nyquist plots of XHGS-*n*. It can be seen that the real part (*Z*′) corresponds to the equivalent of ohmic resistance and the imaginary part (*Z*″) reflects the presence of non-resistive elements. All the samples have the same EIS performances except for XHGS-373 due to its poor electrical conductivity. For high frequencies, the supercapacitors are mainly resistive. With lowering the frequency, Nyquist plots exhibit “Warburg like” behavior (insert of Fig. 8a) which traduces the ion penetration in the thickness of the porous structure of the electrode. At low frequencies, the vertical shape traduces a pure capacitor like-behavior. By comparing the individual patterns in the insets of Fig. 8a, the radius of semicircle decrease with the increase of the heating temperature, indicating that the internal resistance of the XHGS-*n* electrodes decrease with increase of the heating temperature, and also the increase of the electric conductivity in other words.

The impedance frequency behavior was studied using the impedance according to Eqs. (5) and (6):

5 $$C^{{}^{\prime }}\left(\omega \right)=\frac{-Z^{{}^{\prime \prime }}\left(\omega \right)}{{\omega |Z\left(\omega \right)|}^{2}},$$

6 $$C^{{}^{\prime \prime }}\left(\omega \right)=\frac{Z^{{}^{\prime }}\left(\omega \right)}{{\omega |Z\left(\omega \right)|}^{2}},$$

where *C′*(*ω*) is the real part of the capacitance *C*(*ω*) and *C″*(*ω*) is the imaginary part of the capacitance *C*(*ω*). Figure 8b shows the variation of the real part of the capacitance *C* with the frequency. When the frequency is increased, the capacitance decreases and at high frequency the supercapacitor behaves like a pure resistance. Figure 8c shows the variation of the impedance part of the capacitance *C* with the frequency. From Fig. 8c, it is possible to deduce the relaxation time which can be deduced from the frequency *f*_0_ with *τ*_0_ = l/*f*_0_, *f*_0_ can be obtained from the imaginary capacitance plot where it corresponds to the peak frequency. Figure 8d displays the variation of the relaxation time with the heating temperature. From Fig. 8d, the variation of the relaxation time with the heating temperature is the same as specific capacity.

## Conclusions

A series of GSs were prepared by changing the thermal reduction temperature during the reduction of GO to GSs by thermal exfoliation method. The effect of the thermal reduction temperature on the structure and supercapacitive performance of GSs has been investigated. The results show that the GSs with pore sizes center around 4.0 nm. With the increase of the thermal reduction temperature, the number of stacking layers and the structure disorder degree increase, while oxygen-containing group content, the specific surface area, and electrical resistivity of GSs decrease. The results indicate that 673 K is the preferable thermal exfoliation temperature to acquire good supercapacitive performance. In this case, the GSs have the best electrochemical performance (233.1 F g^−1^) in a 6 mol L^−1^ KOH electrolyte.
